# Unravelling a *KMT2A::ARHGEF12* fusion within chromoanagenesis in acute myeloid leukemia using Optical Genome Mapping

**DOI:** 10.1007/s00277-024-05948-w

**Published:** 2024-08-29

**Authors:** Corentine Klos, Pauline Roynard, Céline Berthon, Valérie Soenen, Alice Marceau, Elise Fournier, Laurene Fenwarth, Agnès Daudignon, Catherine Roche, Hélène Guermouche

**Affiliations:** 1 CHU Lille, Institute of Medical Genetics, Jeanne de Flandre Hospital, Avenue Eugène Avinée, 59037 Lille Cedex, France; 2 Hematology Department, CHU Lille, Lille, France; 3 Plasticity and Resistance to Therapies, University of Lille, CNRS, Inserm, CHU Lille, IRCL, UMR9020 – UMR1277 - Canther – Cancer Heterogeneity, Lille, France; 4 Laboratory of Hematology, CHU Lille, Lille, France; 5 Laboratory of Hematology, CH Valenciennes, Valenciennes, France

**Keywords:** Optical genome mapping, Acute myeloid leukemia, *KMT2A::ARHGEF12*, Chromoanagenesis

Dear Editor

An 77-year-old man was referred to our department because of altered general condition for a week, without abnormalities on clinical examination. A blood count showed: white blood cells (WBC) 13.3 × 10^9^/L, haemoglobin concentration 129 g/L and platelet count 30 × 10^9^/L. Examination of WBC differential showed neutropenia (0.8 × 10^9^/L neutrophils), monocytosis (5.8 × 10^9^/L), 1% granulocyte precursors and 34% blasts, eosinophils and lymphocytes being qualitatively and quantitatively normal (respectively 0.1 × 10^9^/L and 1.7 × 10^9^/L). A bone marrow sample was taken and revealed 72% monoblastic blasts, expressing HLA-DR, CD117, CD33, CD11B, CD36 and CD64 by flow cytometry, leading to the diagnosis of acute monocytic leukemia. The karyotype revealed a complex and monosomal clone characterized by an abnormal long arm of chromosome 11 with additional material of unknown origin, a 12p deletion, a loss of chromosome 22 and Y, and a ring chromosome (Fig. 1A). *KMT2A* FISH probe identified an amplification of the 5'*KMT2A* probe (6 copies) on the ring chromosome and normal pattern on both chromosomes 11 (Fig. 1B). The 12p deletion was confirmed with the detection of a monoallelic loss of *ETV6* (data not shown). Detection of fusion transcript by reverse transcriptase multiplex ligation-dependant probe amplification (RT-MPLA) and mutation of *FLT3*, *CEBPA* or *NPM1* were both negative. High throughput DNA sequencing (HTS) using Twist panel on NovaSeq (Illumina) followed by Vardict and MuTect2 variant calling identified mutations of *SF3B1* (p.K666N, VAF 1%) and *TP53* (p.L194P, VAF 40% and p.C238X, VAF 41%).

**Fig. 1:**
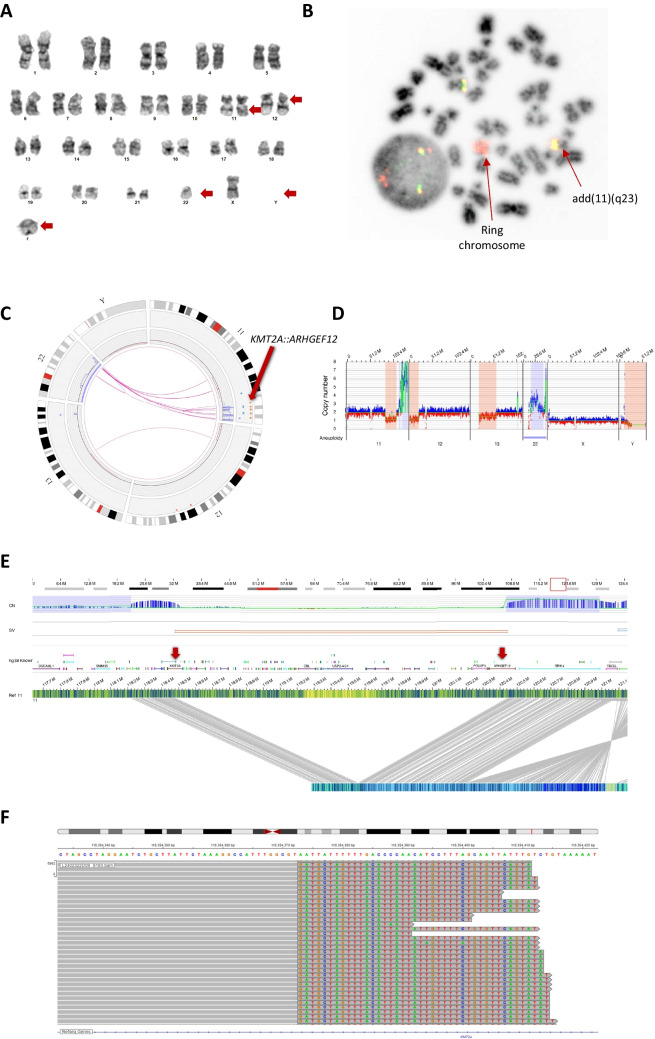
Detection of *KMT2A::ARHGEF12* fusion and its structural mechanism. **A** Medullar karyotype showing the AML clone with an abnormal long arm of chromosome 11, a deletion of the short arm of a chromosome 12, a loss of chromosome 22 and Y, and a ring chromosome. **B** FISH analysis using XL KMT2A Break-Apart METASYSTEMS probe showing a ring chromosome carrying the amplification of the 5'*KMT2A* probe. **C** OGM circos plot displaying chromosomes 11, 12, 13, 22 and Y, *KMT2A::ARHGEF12* fusion is indicated with a red arrow. **D** Status of copy number regarding chromosomes 11, 12, 13, 22, X and Y. **E** OGM molecule carrying the *KMT2A::ARHGEF12* fusion. **F** DNA sequence of *KMT2A* exon 8 (NM_001197104.2) fused to *ARHGEF12* exon 13 (NM_001198665.2), the GT junction being common

Optical Genome Mapping (OGM) revealed 3 major anomalies (Fig. 1C-E). The first was a cryptic deletion of 1.9 Mb within a highly rearranged 11q region leading to a *5'KMT2A::3'ARHGEF12* fusion gene **(**Fig. 1E**)**. The second was a complex event suggesting chromoanagenesis between the 11q and 22q regions with numerous rearrangements and copy number variations, leading to the amplification of the *KMT2A::ARHGEF12* fusion gene (up to 8 copies) consistent with FISH data. Finally, an unbalanced translocation was detected between 12p12.3 and 13q21.1 resulting in partial 12p (including *ETV6*) and 13q deletions (Fig. 1C-D). HTS confirmed the fusion between exon 8 of *KMT2A* and exon 13 of *ARHGEF12* (Fig. 1F). Overall, the cytogenetic data were compatible with the diagnosis of acute myeloid leukemia (AML) with *KMT2A* rearrangement according to WHO classification [1]. Hydroxyurea was started on admission, combined with transfusions of frozen plasma and platelets. Unfortunately, the patient died 3 days after diagnosis.

*KMT2A::ARHGEF12* is an extremely rare fusion with only 8 patients reported in the literature (4 AML, 3 acute lymphoblastic leukemias and 1 high grade B cell lymphoma) and seems to correlate with therapy-related hematological malignancies with poor prognosis [2, 3]. Although more than a hundred fusion partners are described for *KMT2A*, only few of them are recurrent and therefore screened in routine laboratories. However detecting these fusions are crucial, especially as targeted therapies are currently under development [4]. In the present case, usual cytogenetic and RT-MPLA analyses failed to identify this fusion, illustrating the need to improve genetic exploration using fast and pangenomic technologies [5]. The complexity of the 11q rearrangements masked the *KMT2A* fusion partner, *ARHGEF12* which was easily highlighted by OGM. This case clearly demonstrates the strength of this technology for the detection of classifying events of therapeutic value despite the presence of complex rearrangements.

## Data Availability

No datasets were generated or analysed during the current study.
